# Structural Characterization and Biological Evaluation of a Novel GlcNAc-Bearing Exopolysaccharide from the Mangrove Endophytic Fungus *Penicillium janthinellum* N29

**DOI:** 10.3390/md24080290

**Published:** 2026-08-21

**Authors:** Zhuling Shao, Luya Wang, Xin Wang

**Affiliations:** School of Food and Biological Engineering, Yantai Institute of Technology, Yantai 264003, China; 19511527969@163.com (L.W.); lize13053981165@163.com (X.W.)

**Keywords:** marine fungus, exopolysaccharide, structure, insulin resistance, antioxidant activity

## Abstract

A novel, uniform acid heteropolysaccharide with a molecular weight of 13.14 kDa was isolated from the fermented broth of the mangrove endophytic fungus *Penicillium janthinellum* N29, designated as PJ3-1. A structural analysis showed that PJ3-1 is a straight-chain heteropolysaccharide containing GlcN, GlcNAc, Glc and GlcA in the ratio of 12.23:1.79:10.93:1.00. PJ3-1 markedly inhibits α-glucosidase activity and relieves insulin resistance in HepG2 cells. It alleviates lipid peroxidation and upregulates the activities of endogenous antioxidant enzymes. In addition, it also significantly scavenges hydroxyl radicals, ferrous ions, superoxide anions, DPPH radicals and ABTS radicals and enhances reducing power in a dose-dependent manner. Collectively, these findings suggest that PJ3-1 has the potential to serve as a natural antioxidant and hypoglycemic agent for functional food or pharmaceutical applications.

## 1. Introduction

Natural polysaccharides, as essential biopolymers composed of monosaccharide residue linked by glycosidic bonds, have attracted considerable attention due to their diverse biological functions and favorable safety profiles [1]. Accumulating evidence demonstrates that polysaccharides exhibit a broad spectrum of bioactivities, including immunomodulatory, antioxidant, hypoglycemic, and antitumor properties, among which antioxidant capacity is regarded as a fundamental contributor to many of their pharmacological effects [2]. In particular, polysaccharides with strong scavenging activities against DPPH, ABTS, superoxide anion, and hydroxyl radicals have shown promising therapeutic potential for oxidative-stress-related disorders, such as cancer, diabetes, and Alzheimer’s disease [3].

Type 2 diabetes mellitus is a global metabolic disorder characterized by postprandial hyperglycemia. Currently, several α-glucosidase inhibitors, including acarbose, deoxynojirimycin, and voglibose, are clinically used to delay carbohydrate digestion and control blood glucose levels [4]. However, the long-term use of these synthetic agents is often associated with undesirable gastrointestinal side effects, driving an urgent demand for safer and more tolerable alternatives from natural sources [5]. Consequently, natural polysaccharides derived from plants, animals, and microorganisms have emerged as attractive candidates for hypoglycemic agent development, owing to their demonstrated α-glucosidase inhibitory potential and low toxicity.

Compared with polysaccharides derived from plants and animals, microbial polysaccharides, particularly exopolysaccharides (EPSs), secreted extracellularly offer distinct advantages including scalable production, consistent quality, and structural diversity [6]. Microbial EPSs play crucial physiological roles in protecting cells against desiccation, predation, and environmental stress, as well as in facilitating nutrient uptake [7]. Numerous studies have documented the structural diversity and bioactivities of EPSs from various microbial sources [8,9]. Nevertheless, EPSs from mangrove endophytic fungi remain largely underexplored, especially regarding their antioxidant potential and α-glucosidase inhibitory effects.

Mangrove ecosystems are unique intertidal habitats characterized by high salinity, low oxygen, and nutrient fluctuations, which confer exceptional metabolic diversity to their resident endophytic fungi [10]. As the second-largest ecological group of marine-derived fungi, mangrove fungi represent a promising and underexploited resource for discovering novel bioactive natural products. However, the structural features and biological activities of EPSs produced by mangrove endophytic fungi remain poorly characterized, and few studies have systematically investigated the relationship between their structural properties and biological functions.

In the present study, a uniform EPS, designated PJ3-1, was isolated and purified from the fermentation broth of *Penicillium janthinellum* N29, an endophytic fungus obtained from mangrove habitats. Its detailed structural characteristics, including molecular weight, monosaccharide composition, glycosidic linkage patterns, and NMR spectral features, were comprehensively elucidated. Furthermore, the α-glucosidase-inhibitory activity, insulin-sensitizing effect, and antioxidant capacity of PJ3-1 were evaluated in vitro, and a preliminary structure–activity relationship analysis was performed.

## 2. Results and Discussion

### 2.1. Physicochemical Properties and Structure of PJ3-1

**Figure 1:**
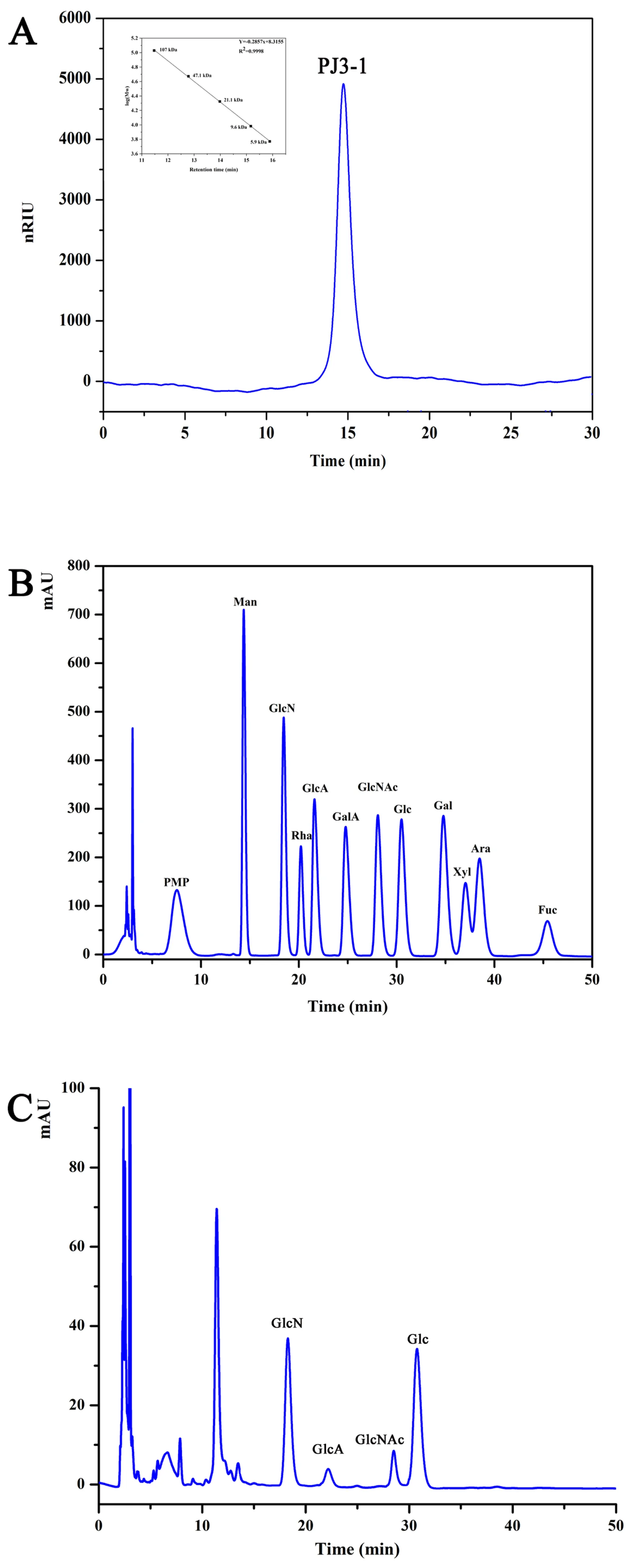
High-performance gel permeation chromatography and high-performance liquid chromatography chromatograms of PJ3-1: (**A**) high-performance gel permeation chromatography chromatogram on a Shodex OHpak SB-803 HQ column and the standard curve of the molecular weight; (**B**) high-performance liquid chromatography chromatogram for the monosaccharide composition analysis (Man: d-mannose, GlcN: d-glucosamine, Rha: l-rhamnose, GlcA: d-glucuronic acid, GalA: d-galacturonic acid, Glc: d-glucose, Gal: d-galactose, Xyl: d-xylose, Ara: l-arabinose, Fuc: l-fucose); (**C**) monosaccharide composition of PJ3-1.

By filtration, concentration, and alcohol precipitation, the crude exopolysaccharide (PJ) was obtained, which was then separated into three fractions using a Q Sepharose Fast Flow column. The third fraction, PJ3, eluted with 0.4 mol/L NaCl, was further purified using a Sephacryl S-100/HR column to obtain PJ3-1, which exhibited a relative uniform molecular weight distribution, characterized by a single, symmetrical peak with a narrow distribution. The molecular weight of PJ3-1 was determined to be 13.14 kDa based on a calibration curve made with pullulan standards (Figure 1A). Analysis of the chemical composition of PJ3-1 revealed that it contained 48.55% neutral sugar, determined by the phenol sulfuric acid method, 0.15% uronic acid, based on the sulfuric acid–carbazole method, and no detectable proteins, using the BCA protein assay kit. The monosaccharide composition analysis, according to the retention times of monosaccharide standards in chromatograms (Figure 1B), indicated that PJ3-1 consisted of GlcN, GlcNAc, Glc, and GlcA in the ratio of 12.23:1.79:10.93:1.0 (Figure 1C).

After successive methylation, hydrolysis, reduction and acetylation, the completeness of methylation of PJ3-1 was confirmed by IR spectroscopy as the disappearance of -OH bands (Supplemental Figure S1). Total ion current chromatograms of PJ3-1 on GC-MS are shown in Supplemental Figure S2. PMAAs derived from PJ3-1 were detected by GC–MS, and the results designated the glycosidic linkage patterns as →2)-Glc*p*-(1→, →4)-Glc*p*N-(1→, →6)-Glc*p*N-(1→ by comparing the mass spectra (CCRC). It was noted that acetyl groups in Glc*p*NAc are removed under strong acidic conditions [11]. The methylated alditol acetates and their respective molar ratios are listed in Table 1. The mass spectra of the individual peaks are shown in Supplemental Figure S3.

**Figure 2:**
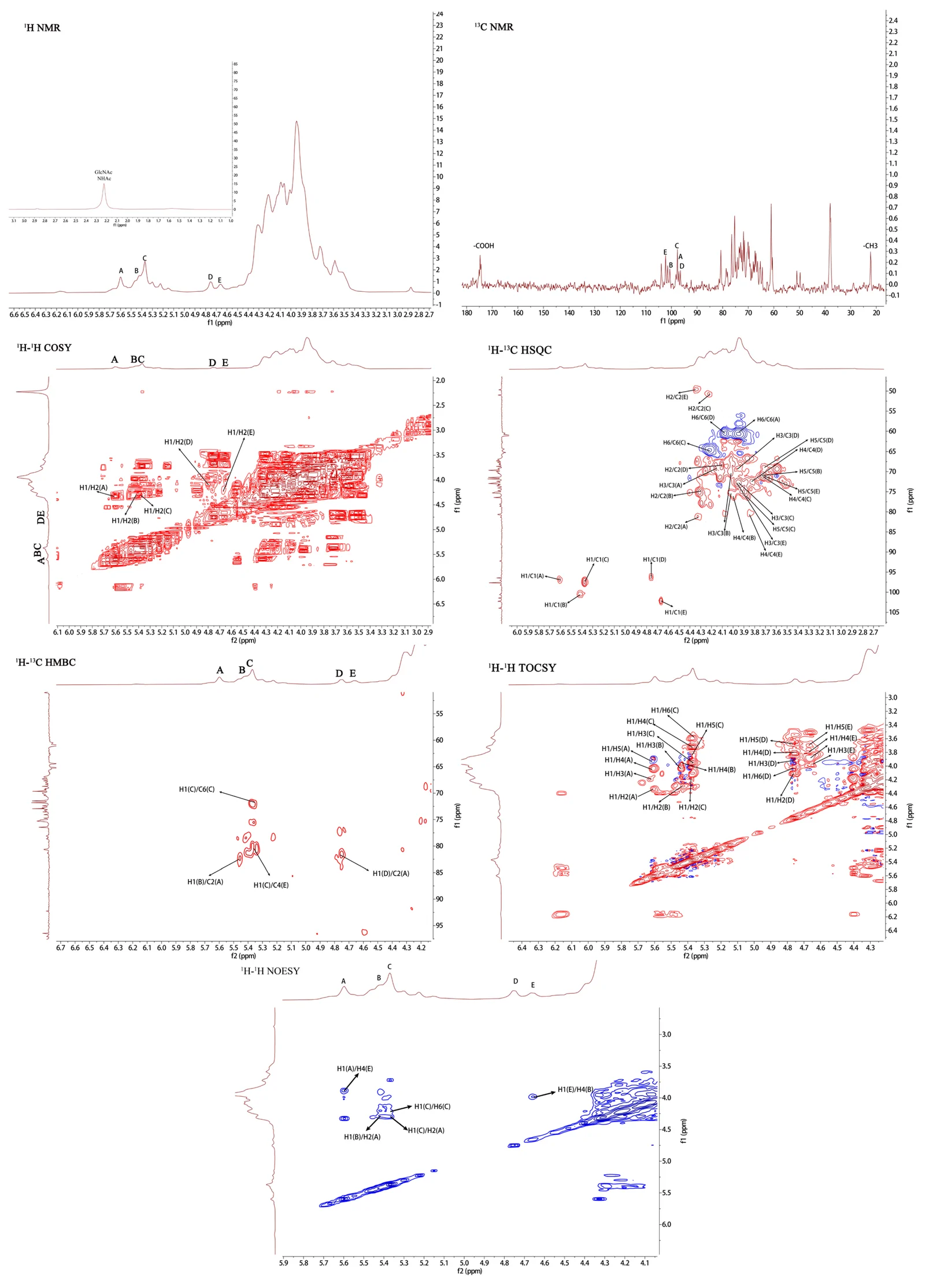
NMR spectra of PJ3-1. Spectra were performed on an Agilent DD2 500 MHz NMR spectrometer using acetone as the internal standard: (A) →2)-α-D-Glc*p*-(1→; (B) →4)-α-D-Glc*p*A-(1→; (C) →6)-α-D-Glc*p*NAc-(1→; (D) β-D-Glc*p*-(1→; (E) →4)-β-D-Glc*p*N-(1→. Glc*p*: glucopyranose.

The structural features of PJ3-1 were further analyzed by 1D/2D NMR spectroscopy (Figure 2 and Figure S4). The ^1^H NMR spectrum of PJ3-1 displayed characteristic anomeric proton signals at 5.60, 5.42, 5.37, 4.76 and 4.66 [12], in addition to other signals in the regions ranging from 2.82 to 4.30 ppm, which referred to H2–H6 of the sugar residues. Amongst them, the proton resonances at 5.5 ppm belonged to the α-anomer, while the protons at 4.7 ppm belonged to the β-anomer [13]. In the ^13^C NMR spectrum, corresponding signals at 96.4, 100.6, 97.4, 96.3 and 102.0 ppm are attributed to anomeric carbons. It is easy to assign the carbons occurring at 174.5–175.0 ppm to -COOH of uronic acid or acetyl carbonyls and the carbons at 22.3 ppm, showing the characteristic carbon signal of the methyl group (-CH_3_) from the N-acetylglucosamine residues [3]. In addition, the signals at 50.8 and 49.4 ppm were related to the C-2 of →6)-Glc*p*NAc-(1→ and →4)-Glc*p*N-(1→, and the remaining carbons were distributed in the region of 60.9–80.7 ppm.

The ^1^H-H COSY and ^1^H-^1^H TOCSY spectra facilitated the identification of proton correlations within the sugar residues. The ^1^H-^13^C correlations were tentatively assigned from the HSQC spectrum. The proton signal of residue A at 5.60 ppm was correlated with the carbon at 96.4 ppm, which was assigned to the →2)-α-D-Glc*p*-(1→ residue due to the downfield shift of C-2 compared with the parent α-D-Glc*p* residue [14,15,16]. The proton at 5.42 ppm and the carbon at 100.6 ppm of residue B were correlated, belonging to the →4)-α-D-Glc*p*A-(1→ residue, supported by the downfield chemical shift of C-4 at 76.3 ppm because of the presence of a glycosidic substitution at the C4 hydroxyl position of the pyranose ring [16,17,18]. The H-1 signal of residue C at 5.37 ppm was correlated with the C-1 signal at 97.4 ppm, and the carbon signal at 50.8 ppm was assigned to C-2 of residue C. This residue was identified as the →6)-α-D-Glc*p*NAc-(1→ residue due to the low displacement shift of C-6 [19,20,21]. The H-1 signal of residue D at 4.76 ppm was correlated with the C-1 signal at 96.3 ppm, and residue D was identified as the β-D-Glc*p*-(1→ [22]. The H-1 signal of residue E at 4.66 ppm was correlated with the C-1 signal at 102.0 ppm, and residue E was assigned to the →4)-β-D-Glc*p*N-(1→ due to the correlated H-4/C-4 signals (3.84/80.1 ppm) [23,24,25]. By integrating data from the ^1^H-^1^H COSY, TOCSY, and HSQC spectra, we accurately assigned the primary proton and carbon signals associated with the five sugar residues, as summarized in Table 2.

**Figure 3:**
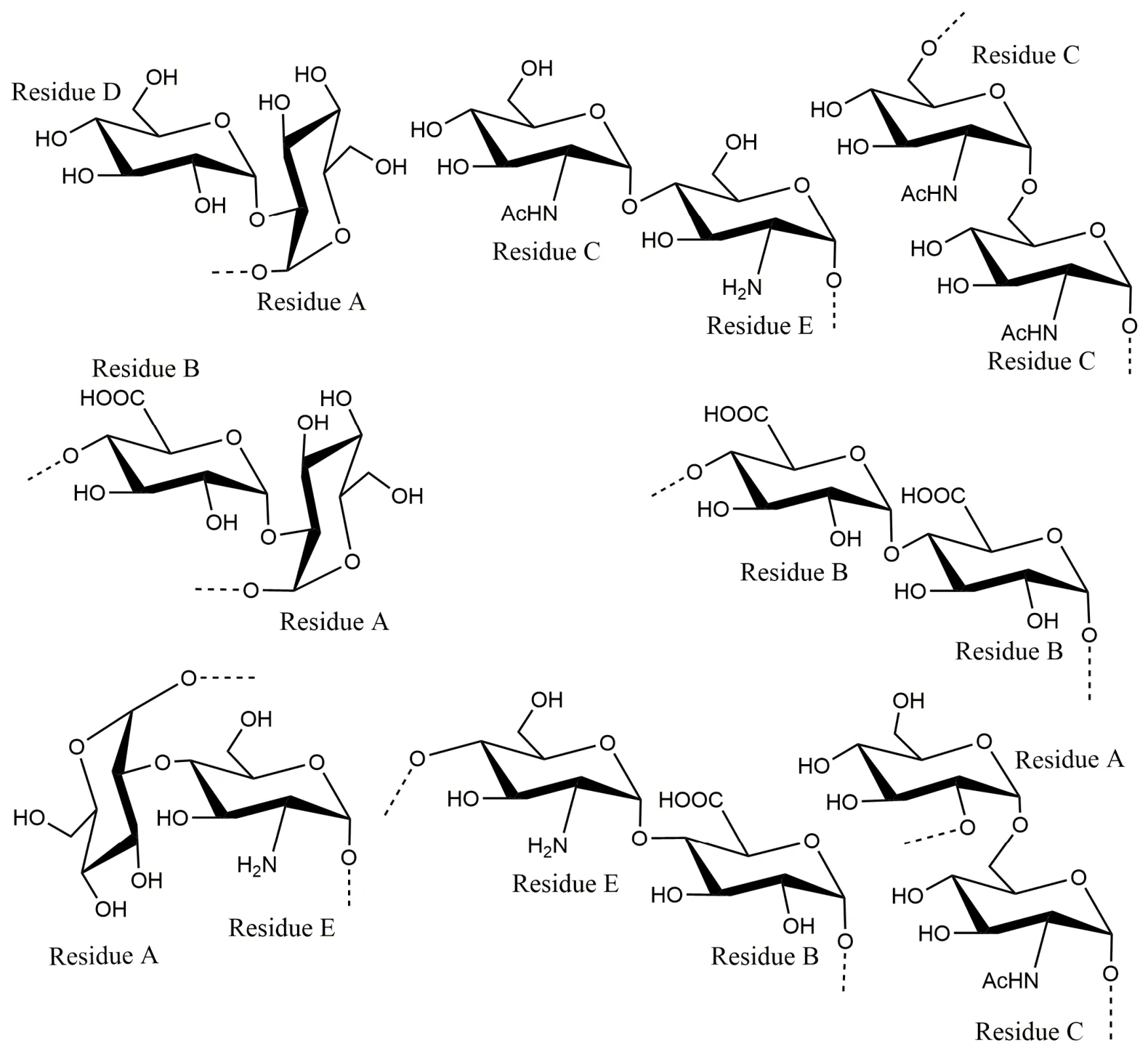
Main disaccharide fragments in PJ3-1.

The repetitive sequences observed in PJ3-1 were determined by HMBC and NOESY. The cross signals H-1(D)/C-2(A) in HMBC indicated that the C-1 of the β-D-Glc*p*-(1→ residue was linked to the O-2 position of the →2)-α-D-Glc*p*-(1→ residue. The cross signals H-1(C)/C-4(E) showed the presence of →6)-α-D-Glc*p*NAc-(1→4)-β-D-Glc*p*N-(1→ residues. The H-1 (5.37 ppm) of the →6)-α-D-Glc*p*NAc-(1→ residue was related to the C-6 of the →6)-α-D-Glc*p*NAc-(1→ residue and cross signals H1(C)/H6(C) in NOESY, indicating the existence of fragment →6)-α-D-Glc*p*NAc-(1→6)-α-D-Glc*p*NAc-(1→ residues. Cross signals H-1(B)/C-2(A) in HMBC and H1(B)/H2(A) in NOESY proved that the C-1 of the →4)-α-D-Glc*p*A-(1→ residue was linked to the O-2 position of the →2)-α-D-Glc*p*-(1→ residue. Cross signals H-1(B)/C-4(B) in HMBC and H1(B)/H4(B) in NOESY proved that the C-1 of the →4)-α-D-Glc*p*A-(1→ residue was linked to the O-4 position of the →4)-α-D-Glc*p*A-(1→ residue. The cross signals H1 (A)/H4(E) in NOESY showed the presence of →2)-α-D-Glc*p*-(1→4)-β-D-Glc*p*N-(1→ residues. The cross signals H1 (C)/H2(A) in NOESY showed the presence of →6)-α-D-Glc*p*NAc-(1→2)-α-D-Glc*p*-(1→ residues. The cross signals H1 (E)/H4(B) in NOESY showed the presence of →4)-β-D-Glc*p*N-(1→4)-α-D-Glc*p*A-(1→. The above analysis indicates that PJ3-1 is a straight-chain heteropolysaccharide that is constituted by the β-D-Glc*p*-(1→2)-α-D-Glc*p*-(1→, →6)-α-D-Glc*p*NAc-(1→4)-β-D-Glc*p*N-(1→, →6)-α-D-Glc*p*NAc-(1→4)-β-D-Glc*p*N-(1→, →4)-α-D-Glc*p*A-(1→2)-α-D-Glc*p*-(1→, →4)-α-D-Glc*p*A-(1→4)-α-D-Glc*p*A-(1→, →2)-α-D-Glc*p*-(1→4)-β-D-Glc*p*N-(1→, →6)-α-D-Glc*p*NAc-(1→2)-α-D-Glc*p*-(1→ and →4)-β-D-Glc*p*N-(1→4)-α-D-Glc*p*A-(1→ residues. The deduced main disaccharide fragments of PJ3-1 are illustrated in Figure 3. These structures were deduced from a comprehensive analysis of the cross signals observed from the NMR spectra, providing insight into the repeating residues within the polysaccharide chain.

To date, there is limited documentation on the structural characterization of the exopolysaccharides from mangrove endophytic fungi. Natural polysaccharide “chitin” is a well-known glycosaminoglycan characterized by its β-1,6-linked long GlcNAc polymer, which exhibits a diverse range of biological activities [25]. A novel heteropolysaccharide, named GSPB70-S, was isolated and purified from the fruit body of *G. sinense*. Its backbone composition includes →3)-β-D-Glc*p*-(1→4)-α-D-Glc*p*NAc-(1→4)-α-D-Man*p*-(1→3)-β-D-Glc*p*-(1→, with side chains consisting of β-D-Glc*p*-(1→, α-D-Glc*p*NAc-(1→ and →4)-α-D-Gal*p*-(1→ [26]. GSPB70-S exhibits robust immunomodulatory effects, such as enhancing macrophage proliferation, activating T and B lymphocytes, as well as natural killer cells, and stimulating the release of cytokines and antibodies. These properties underscore its potential as a powerful biological agent in immune-system regulation.

Another novel polysaccharide, MIPW50-1, extracted from *M. importuna* fruiting bodies, is composed of →2,3,6)-α-D-Man*p*-(1→, →3,6)-α-D-Man*p*-(1→, →2)-α-D-Gal*p*-(1→, →6)-α-D-Man*p*-(1→, →4)-β-D-Glc*p*-(1→, →4)-α-D-Glc*p*NAc-(1→, →6)-α-D-Glc*p*-(1→, α-D-Gal*p*-(1→, α-D-Glc*p*NAc-(1→, and β-D-Glc*p*-(1→. MIPW50-1 significantly enhances the phagocytic function of macrophages and promotes the accumulation of nitric oxide (NO), tumor necrosis factor-alpha (TNF-α), and interleukin-6 (IL-6). These activities highlight its potential in modulating immune responses and contributing to the body’s defense mechanisms [27]. These findings demonstrate that polysaccharides containing GlcNAc, isolated from both fungi and bacteria, exhibit considerable biological activity. However, the biological activity of exopolysaccharides containing GlcNAc from mangrove endophytic fungi still requires further investigation.

### 2.2. Antioxidant Activity of PJ3-1 In Vitro

#### 2.2.1. Scavenging DPPH Radical Activity

**Figure 4:**
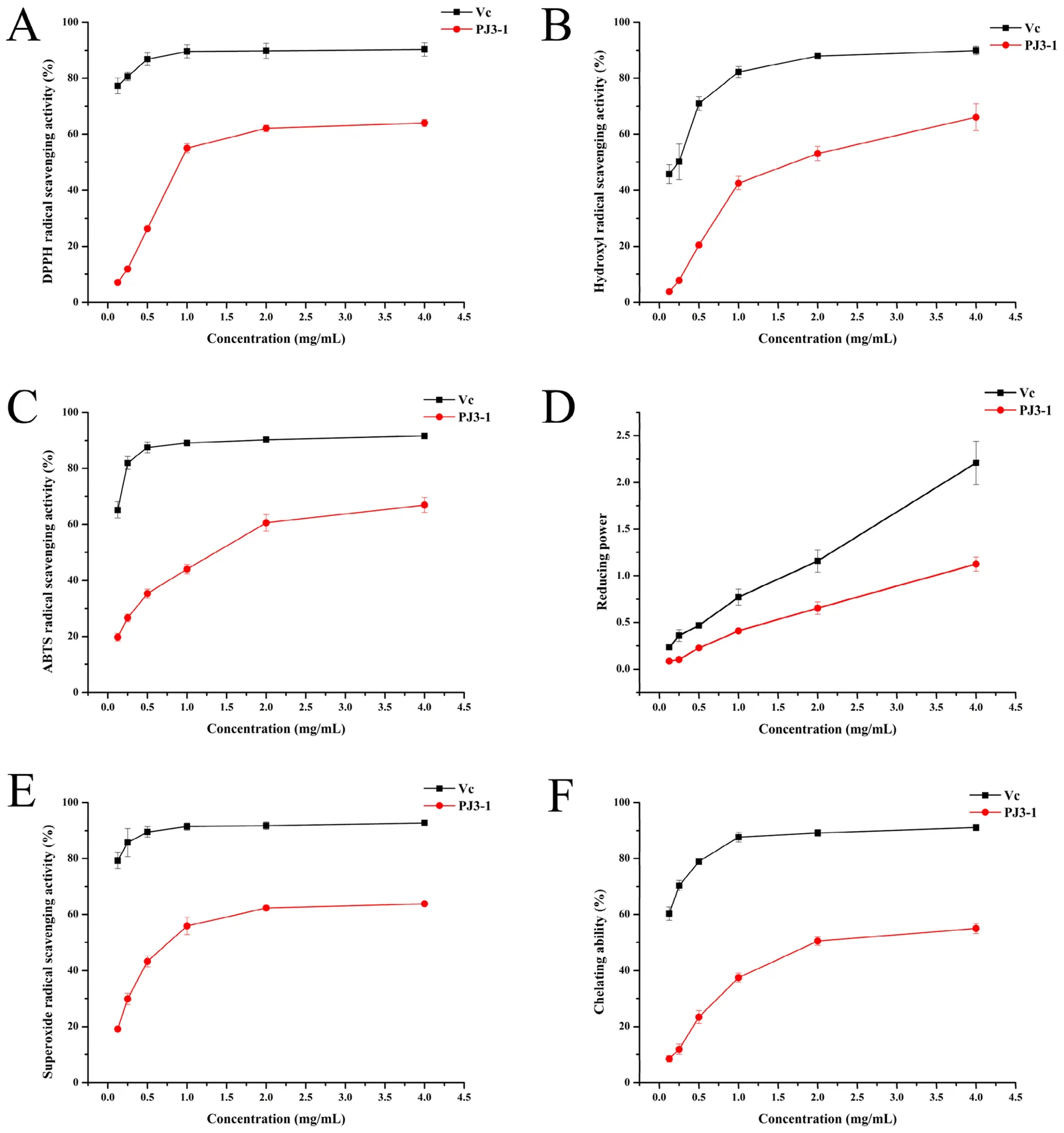
Determination of the antioxidant activities of PJ3-1: (**A**) scavenging DPPH radical activity; (**B**) hydroxyl radical scavenging activity; (**C**) ABTS radical scavenging activity; (**D**) reducing power assay; (**E**) scavenging ability of superoxide anion; (**F**) Fe^2+^ chelating ability. Data are expressed as the mean ± SD, *n* = 3.

DPPH radical activity is widely used to assess the antioxidant capacity of drugs and foods. DPPH in absolute ethanol solution exhibits maximum absorbance at 517 nm. When exposed to an electron- or hydrogen-donating substance, such as an antioxidant, DPPH is scavenged, leading to a reduction in absorbance at 517 nm. This decrease in absorbance indicates the scavenging ability of the substance being tested, providing a measure of its antioxidant potential. The DPPH scavenging activity of the sample is inversely proportional to the reaction mixture’s absorbance at 517 nm. That is, as the absorbance decreased, the scavenging activity increased [28]. The scavenging DPPH activities of PJ3-1 are presented in Figure 4A. Ascorbic acid was used as the positive control. The results show that both PJ3-1 and ascorbic acid displayed notable DPPH radical scavenging activity, which increased with the concentration. At concentrations ranging from 0.125 to 4.0 mg/mL, the scavenging rate of PJ3-1 rose from 7.18 ± 0.24% to 64.03 ± 1.27%. The median effect dose (EC_50_) values for the scavenging DPPH radical was 1.33 mg/mL for PJ3-1. PJ3-1 exhibited enhanced scavenging activity, albeit less potent than ascorbic acid. Similar findings have been observed in other polysaccharides [29,30].

#### 2.2.2. Hydroxyl Radical Scavenging Activity

The hydroxyl radical, a type of reactive oxygen species, can cause significant oxidative damage in cells. This includes disrupting key biomolecules, like proteins, lipids, and DNA, and impairing cell membrane permeability. These processes can cause tissue damage and, ultimately, lead to cell death [31]. Therefore, scavenging hydroxyl radicals is crucial for protecting living systems. Figure 4B illustrates the hydroxyl radical scavenging ability of PJ3-1 at various concentrations, with ascorbic acid serving as the positive control. At a concentration of 4.0 mg/mL, PJ3-1 achieved its highest hydroxyl radical scavenging rate of 66.11 ± 4.87%. The results suggest that PJ3-1 has the capacity to scavenge hydroxyl radicals. The potential mechanism may involve polysaccharides providing either an electron or a hydrogen atom, which interacts with hydroxyl radicals to form a stable compound, thereby terminating the chain reaction [32].

#### 2.2.3. ABTS Radical Scavenging Activity

The ABTS assay is a widely recognized method for measuring the total antioxidant capacity of a substance. Figure 4C displays the scavenging ability of PJ3-1 and ascorbic acid on ABTS free radicals, illustrating their effectiveness in neutralizing these radicals. The scavenging efficacy of PJ3-1 exhibited a strong correlation with increasing concentrations, albeit slightly inferior to that of ascorbic acid at equivalent concentrations. At the concentration of 4 mg/mL, the ABTS radical scavenging rate of PJ3-1 was 66.96 ± 2.64%. These results suggest that PJ3-1 possesses the ability to scavenge ABTS radicals, thus positioning it as a promising antioxidant candidate.

#### 2.2.4. Reducing Power Assay

In the reducing power assay, antioxidants facilitate the reduction of the Fe^3+^/ferricyanide complex to its ferrous form (Fe^2+^) through electron donation. The presence of Fe^2+^ is subsequently detected by measuring the formation of Perl’s Prussian blue at 700 nm [33]. Figure 4D presents the reducing power of PJ3-1 and ascorbic acid, indicating that a higher absorbance value reflects a greater reducing power. At a concentration of 4.0 mg/mL, PJ3-1 achieved a maximum absorbance of 1.13 ± 0.074. Antioxidant polysaccharides act as electron donors, capable of interacting with free radicals. The moderate reducing power exhibited by PJ3-1 suggests the presence of free electrons available for reaction with Fe^3+^, although its reducing power was notably less potent compared with ascorbic acid.

#### 2.2.5. Scavenging Ability of Superoxide Anion

Superoxide radicals, considered as initial free radicals, are formed in the mitochondrial electron transport system. They cause cellular damage by harming DNA and membrane lipids, which can lead to lipid peroxidation and trigger pathological conditions such as arthritis and Alzheimer’s disease [34,35]. As depicted in Figure 4E, PJ3-1 exhibited a concentration-dependent scavenging activity on superoxide radicals. The maximum scavenging ability of 63.83 ± 0.49% was observed for PJ3-1 at a concentration of 4.0 mg/mL. However, ascorbic acid showed significantly higher scavenging activity than PJ3-1 across concentrations ranging from 0.125 to 4.0 mg/mL. Polysaccharides with unique conformations can facilitate the liberation of hydrogen in single O-H bonds, thereby potentially stabilizing the superoxide anion. The mechanism by which PJ3-1 scavenges superoxide anions may be related to the dissociation energy of the O-H bond. This lower dissociation energy facilitates the donation of hydrogen atoms to neutralize superoxide radicals, thereby preventing cellular damage.

#### 2.2.6. Fe^2+^ Chelating Ability

The antioxidant potential of polysaccharides can sometimes be enhanced by their ability to chelate metals. The Fe^2+^ chelating capacity of PJ3-1 at varying concentrations is depicted in Figure 4F. PJ3-1 demonstrated considerably weaker metal-chelating activity compared with EDTA at equivalent concentrations. At 4.0 mg/mL, PJ3-1 achieved a chelating rate of 55.08 ± 1.81%, whereas EDTA reached 91.10 ± 1.12%. Research suggests that the chelation of metal ions by polysaccharides may result from the formation of a cross-bridge between uronic acid’s carboxyl group and a divalent ion. Additionally, differences in ferrous ion chelation among various acidic polysaccharides were found to be minimal. The high uronic acid content in PJ3-1 may partly explain its effective Fe^2+^ scavenging activity.

### 2.3. α-Glucosidase-Inhibitory Activity

**Figure 5:**
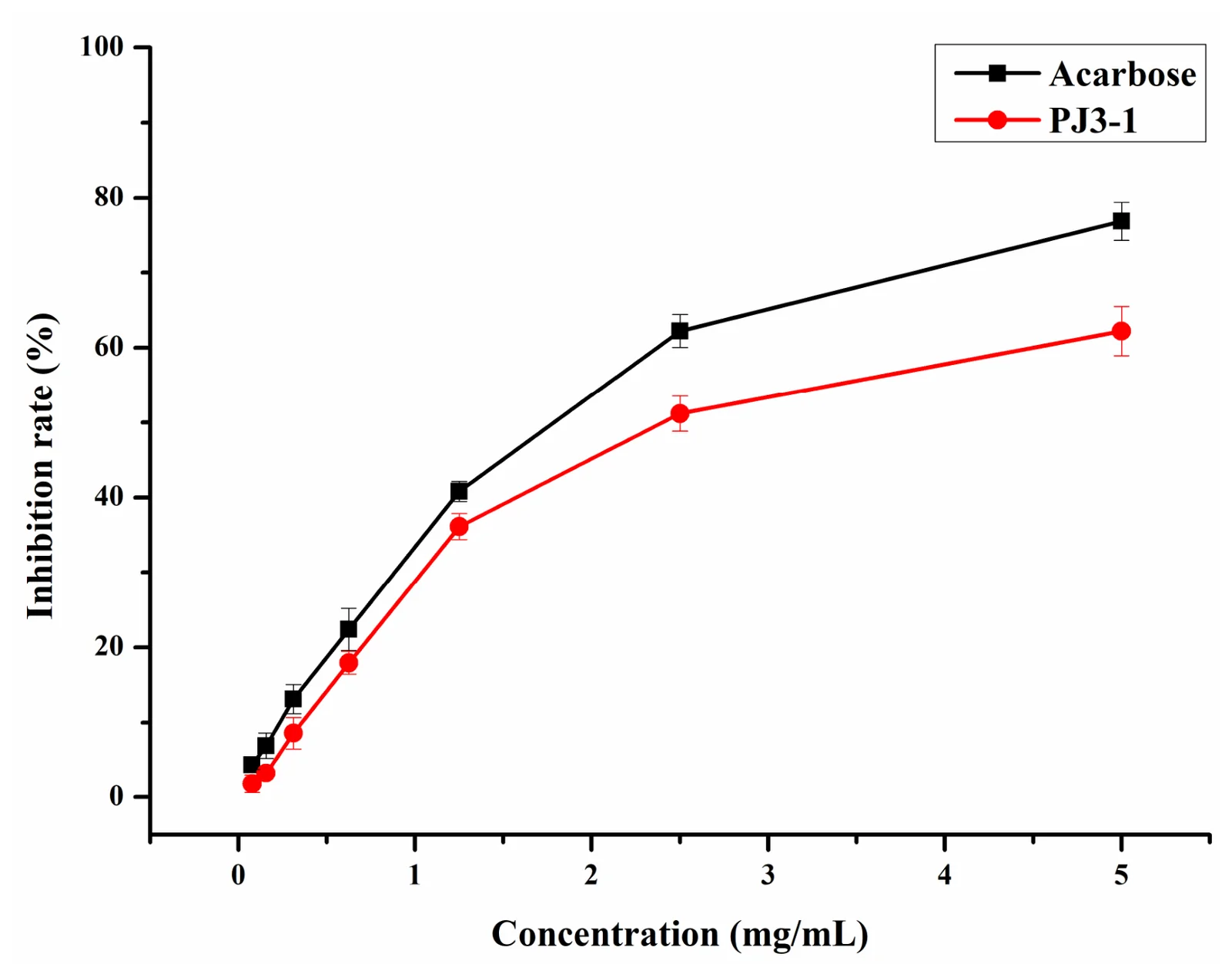
α-Glucosidase-inhibitory activities of PJ3-1 and acarbose. Data are expressed as the mean ± SD, *n* = 3.

As shown in Figure 5, acarbose was used as a positive control to investigate the α-glucosidase-inhibitory activity of PJ3-1. PJ3-1 exhibited inhibitory activity on α-glucosidase across varying concentrations, demonstrating a dose-dependent response. At a concentration of 5 mg/mL, the maximum inhibition rate was 68.21 ± 3.25%. The half-maximal inhibitory efficiency of the inhibitor (IC_50_) value of PJ3-1 was determined to be 2.26 mg/mL. The enhanced inhibitory activity of PJ3-1 on α-glucosidase may be attributed to the presence of the α-(1,4) glycosidic bond in its structure [36]. Reported data indicate a positive correlation between the inhibitory activity of polysaccharides on digestive enzymes and their uronic acid content [37]. This suggests that the structural features of PJ3-1 contribute significantly to its functional properties, potentially making it a valuable candidate for therapeutic applications targeting digestive enzyme regulation. Uronic acid was also detected in PJ3-1, although the content was low. In addition, reported data indicate that a higher content of GlcNAc is also one of the reasons for higher α-glucosidase-inhibitory activity [32]. The combination of these factors has resulted in PJ3-1 exhibiting α-glucosidase-inhibitory activity. Its structural features, including the presence of the α-(1,4) glycosidic bond and uronic acid content, contribute to its potent inhibitory effects, highlighting its potential for therapeutic use in managing conditions such as diabetes.

### 2.4. IR-HepG2 Modeling Concentration Establishment

**Figure 6:**
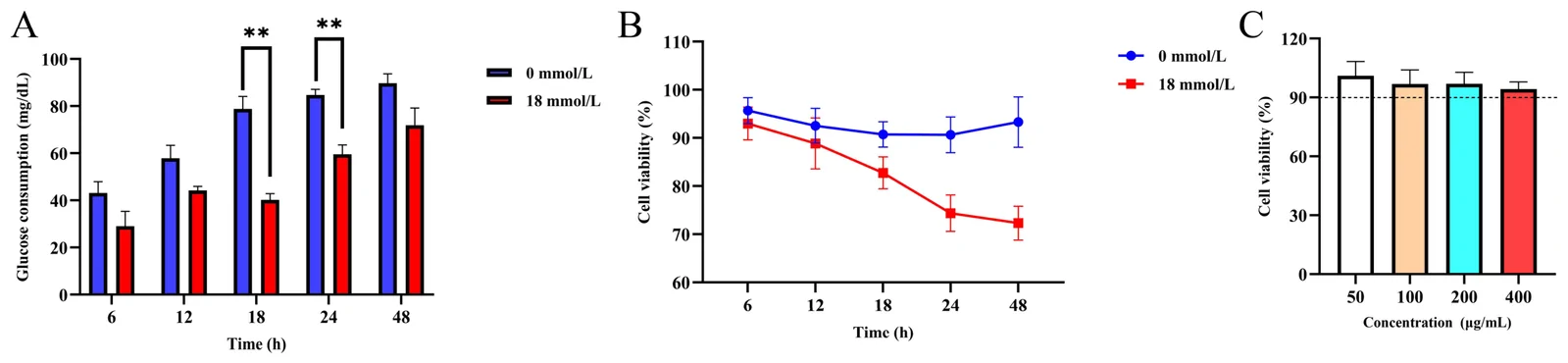
Determination of the induction conditions for IR-HepG2 cells: (**A**) 18 mmol/L glucosamine at different times of glucose consumption (** *p* < 0.01 vs. 0 mmol/L group); (**B**) 18 mmol/L glucosamine at different times for cell viability; (**C**) effect of different concentrations of PJ3-1 on the viability of HepG2 cells. Data are expressed as the mean ± SD, *n* = 3.

In this experiment, glucosamine was utilized to induce insulin resistance in HepG2 cells. As shown in Figure 6A. The results revealed that 18 mmol/L glucosamine markedly suppressed cellular glucose consumption after 18 h and 24 h of treatment relative to the Blank group. Nevertheless, MTT assay verified that glucosamine exhibited elevated cytotoxicity following 24 h of incubation (Figure 6B). Accordingly, 18 mmol/L glucosamine with 18 h of intervention was selected for subsequent experiments to maximize the insulin resistance induction efficacy and minimize adverse cytotoxic effects.

### 2.5. Effect of PJ3-1 on HepG2 Cell Viability

The cytotoxicity of polysaccharide PJ3-1 against HepG2 cells was evaluated by an MTT assay in vitro. As shown in Figure 6C, the viability of HepG2 cells remained above 90% at concentrations ranging from 50 to 400 μg/mL, indicating that PJ3-1 exhibits high biocompatibility under the tested conditions. To achieve optimal effects at the lowest possible concentrations, 50, 100 and 200 μg/mL were selected for the subsequent experiments.

### 2.6. Effect on Glucose Utilization in IR-HepG2 Cells

**Figure 7:**
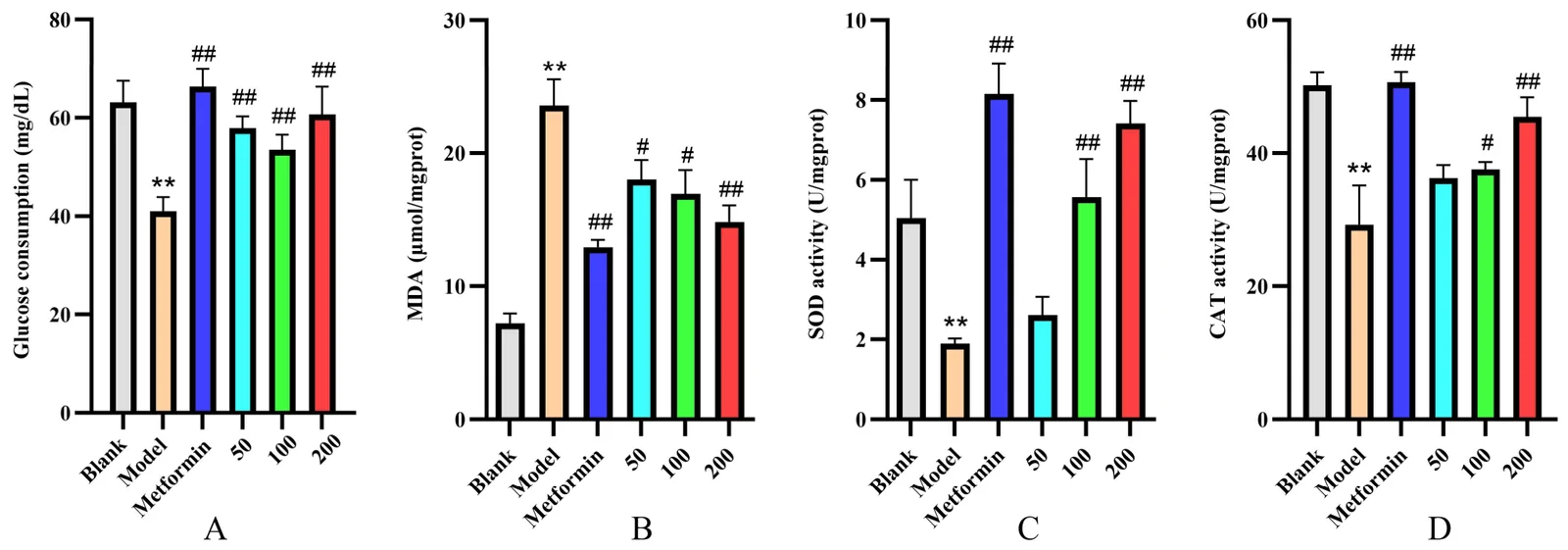
(**A**) Glucose consumption in IR-HepG2 cells after treatment with PJ3-1 (50, 100 and 200 μg/mL) or metformin (200 μg/mL) (** *p* < 0.01 vs. Blank group; ^##^ *p* < 0.01 vs. Model group). (**B**) The levels of malondialdehyde (MDA). (**C**) The activities of superoxide dismutase (SOD) (**D**) and catalase (CAT) in IR-HepG2 cells treated with PJ3-1 (50, 100 and 200 μg/mL) or metformin (200 μg/mL) (** *p* < 0.01 vs. Blank group; ^#^ *p* < 0.05 vs. Model group; ^##^ *p* < 0.01 vs. Model group; data are expressed as the mean ± SD, *n* = 3).

IR-HepG2 cells were treated with different concentrations of polysaccharides or Metformin, and their effects on glucose utilization were investigated. As showed in Figure 7A, the glucose consumption level in the Model group was significantly reduced compared with the Blank group (*p* < 0.01), demonstrating the successful establishment of the insulin resistance model in HepG2 cells. Relative to the Model group, the administration of metformin induced an extremely significant elevation in glucose consumption by IR-HepG2 cells (*p* < 0.01,), confirming the pharmacological sensitivity of the established cell model. Treatment with PJ3-1 at the three concentrations (50, 100, 200 μg/mL) all produced extremely significant enhancements in glucose consumption by the IR-HepG2 cells (*p* < 0.01). The cellular glucose consumption capacity increased gradually with the rising concentration of PJ3-1, indicating a dose-dependent pattern of PJ3-1 in ameliorating insulin resistance and promoting glucose utilization. Specifically, the glucose consumption level at the 200 μg/mL concentration of the PJ3-1 group was comparable to that of the metformin group, revealing a prominent insulin-sensitizing and hypoglycemic effect of high-dose PJ3-1 at the cellular level.

### 2.7. Antioxidant Analysis in IR-HepG2 Cells

Oxidative stress is positively correlated with insulin resistance, which plays a central role in the pathogenesis of type 2 diabetes mellitus. Excessive oxidative stress combined with impaired antioxidant capacity triggers a series of signal transduction cascades, which disrupt insulin receptor function and interfere with glucose transporter trafficking, leading to further elevation of blood glucose levels [38,39]. Hyperglycemia, in turn, induces protein glycosylation, which inactivates antioxidant enzymes such as SOD and CAT, causes dyslipidemia in diabetic patients, and promotes the production of lipid peroxides including malondialdehyde (MDA). Therefore, inhibiting oxidative stress may serve as a potential adjuvant therapy to lower blood glucose and alleviate diabetic symptoms and complications [40,41]. In the present study, the effects of polysaccharide PJ3-1 on oxidative stress in IR-HepG2 cells were evaluated by measuring the activities of SOD and CAT and the level of MDA.

As shown in Figure 7B, compared with the Blank control group, the MDA level in the Model group significantly increased by approximately 227.44% (*p* < 0.01), indicating severe lipid peroxidation and successful establishment of the oxidative stress model. Treatment with metformin markedly reduced the MDA content by 45.21% (*p* < 0.01) relative to the Model group. Notably, PJ3-1 exhibited a clear dose-dependent inhibitory effect on MDA accumulation. At concentrations of 50, 100 and 200 μg/mL, MDA levels decreased by 23.60% (*p* < 0.05), 28.09% (*p* < 0.05) and 37.12% (*p* < 0.01), respectively, compared with the Model group. The effects of PJ3-1 on SOD activity are presented in Figure 7C. The Model group showed a dramatic 62.51% reduction in SOD activity (*p* < 0.01) compared with the Blank control, reflecting a severely impaired antioxidant defense system. PJ3-1 at 50 μg/mL caused a slight increase in SOD activity but without statistical significance. In contrast, treatments with 100 and 200 μg/mL resulted in robust elevations of SOD activity by 194.71% (*p* < 0.01) and 292.06% (*p* < 0.01), respectively, versus the Model group, demonstrating a pronounced dose–response relationship. Consistent with the SOD results, CAT activity in the Model group was significantly suppressed to 41.73% of the Blank control level (*p* < 0.01, Figure 7D). Metformin treatment effectively reversed this suppression, increasing CAT activity by 73.18% (*p* < 0.01) compared with the Model group. PJ3-1 also dose-dependently enhanced CAT activity. While 50 μg/mL showed a mild increase in CAT activity without statistical significance, 100 and 200 μg/mL treatments significantly elevated CAT activity by 28.29% (*p* < 0.05) and 55.38% (*p* < 0.01), respectively, relative to the Model group. Collectively, these data demonstrate that PJ3-1 exerts potent antioxidant effects by attenuating lipid peroxidation and upregulating the activities of endogenous antioxidant enzymes in a dose-dependent manner.

PJ3-1, extracted from the mangrove endophytic fungus *Penicillium janthinellum* N29, comprises GlcN, GlcNAc, Glc, and GlcA residues. Notably, this study is the first to identify GlcNAc in *Penicillium janthinellum* N29. GlcNAc plays a vital role as a fundamental component of fungal cell walls and is essential for the synthesis of the extracellular matrix in animal cells. Polysaccharides that include GlcNAc demonstrate unique bioactivities, notably immunomodulatory effects and hypoglycemic activity [42]. The acetyl group can influence intermolecular forces, altering the height and width of polysaccharides, leading to aggregation and changes in their higher structure, which affects hypoglycemic activity [43]. Acetylglucosamine is a versatile glucosamine involved in the synthesis of numerous biological macromolecules in humans. Moreover, the proportion of monosaccharides is crucial for hypoglycemic activity. Polysaccharides dissolve in water by forming hydrogen bonds; those with higher uronic acid content tend to form more hydrogen bonds. Higher glucuronic acid content results in a more dispersed state, enhancing the hypoglycemic activity of polysaccharides [44]. Two distinct polysaccharides, WSRP-2a and WSRP-2b, were isolated from *Rosa setate* × *Rosa rugosa*. Notably, WSRP-2b exhibited a higher content of glucuronic and galacturonic acids, correlating with enhanced α-amylase and α-glucosidase-inhibitory activities [45]. Therefore, we speculate that glucuronic acid content and GlcNAc contribute to the antioxidant and hypoglycemic activities of PJ3-1.

Furthermore, Zhong et al. demonstrated that FSIPs, polysaccharide fractions extracted from unopened flower buds of *Sophora japonica*, improved glucose disposal in IR-HepG2 cells [46]. Mechanistically, FSIPs attenuated intracellular oxidative stress by decreasing ROS and MDA accumulation while upregulating the enzymatic activities of SOD and CAT, thereby augmenting cellular antioxidant defense systems and mitigating insulin resistance-associated cellular injury. This bioactivity was closely correlated with the abundant GalA residues present in FSIPs. Comparable observations have been reported for polysaccharides isolated from *Coptis chinensis* and *Cordyceps militaris*, which are also characterized by high uronic acid contents. These polysaccharides consistently exhibit the ability to reduce MDA levels and enhance SOD and CAT activities, contributing to their antidiabetic properties [47]. In alignment with our present study, these cumulative findings strongly support the pivotal role of uronic acid in mediating the hypoglycemic effects of polysaccharides.

## 3. Materials and Methods

### 3.1. Materials

P-Nitrophenyl-α-D-glucopyranoside (pNPG), α-glucosidase (EC 3.2.1.20, from Saccharomyces cerevisiae), 1-phenyl-3-methyl-5-pyrazolone (PMP) and monosaccharide standard (D-galactose, D-galacturonic acid, D-glucose, D-glucuronic acid, N-acetyl-β-D-glucosamine, D-mannose, D-xylose, L-arabinose, L-fucose and L-rhamnose) were purchased from Sigma Chemical Co. (St. Louis, MO, USA). Pullulan standards (*Mw*: 5.9, 9.6, 21.1, 47.1 and 107 kDa) were obtained from Showa Denko K.K (Tokyo, Japan). BCA protein assay kit was from Beyotime Biotechnology (Shanghai, China). Q Sepharose^TM^ Fast Flow and Sephacryl^TM^ S-100 medium gel were from GE healthcare Life Science (Piscataway, NJ, USA). 1-Diphenyl-2-picrylhydrazyl (DPPH), nitrotetrazolium blue chloride (NBT), nicotinamide adenine dinucleotide (NADH), 2,2’-azinobis (3-ethylbenzothiazoline-6-sulfonic acid ammonium salt) (ABTS) and metformin were from Aladdin Chemical Co., Ltd. (Shanghai, China). Dulbecco’s modified eagle medium (DMEM), trypsin–EDTA digestive solution, dimethyl sulfoxide, penicillin–streptomycin, and phosphate-buffered saline were obtained from Gibco Company (Grand Island, NY, USA). All other chemicals were analytical-grade commercial preparations.

### 3.2. Preparation of the Exopolysaccharide PJ3-1

A novel exopolysaccharide PJ1-1 has been reported from the fungus *P. janthinellum* N29 in our previous study [48]. As a continuing search for *P. janthinellum* N29, the crude EPS (PJ) was applied to a Q Sepharose Fast Flow column (60 cm × 3 cm) and eluted with 0, 0.025 and 0.4 mol/L NaCl at a flow rate of 1.0 mL/min. The polysaccharide fractions were collected and detected using the phenol–sulfuric acid method to draw the elution curve [49]. Amongst the main fractions, PJ3 was further purified using a Sephacryl S-100 column (95 cm × 2.5 cm), eluted with 0.2 mol/L NH_4_HCO_3_ and then freeze-dried to obtain a novel EPS, PJ3-1.

### 3.3. Chemical Composition and Mw of PJ3-1

The total sugar, protein, and uronic acid contents were determined using the phenol–sulfuric acid method, the BCA protein assay kit, and the sulfuric acid–carbazole method, respectively [50,51]. The purity and molecular weight of PJ3-1 were evaluated via high-performance gel permeation chromatography (HPGPC) conducted on an Agilent 1260 series system (Agilent Technologies, Santa Clara, CA, USA) equipped with a differential refractive index detector (Agilent RID-10A Series) and a Shodex OHpak SB-803 HQ column (8.0 mm × 300 mm, Showa Denko K.K., Tokyo, Japan). The mobile phase was 0.1 mol/L Na_2_SO_4_ with a flow rate of 0.5 mL/min, and the injection volume was 20 μL. Pullulan standards with molecular weights of 5.9, 9.6, 21.1, 47.1, and 107 kDa were used to create molecular-weight standard curves based on the retention time and molecular weight [52].

### 3.4. Monosaccharide Composition

The monosaccharide composition of PJ3-1 was determined using a reversed-phase HPLC method with PMP pre-column derivatization [11]. In brief, 5 mg of PJ3-1 was mixed with 500 μL of 4 mol/L trifluoroacetic acid (TFA), which was then transferred to a sealed ampoule and hydrolyzed for 2.5 h at 140 °C. Upon cooling to room temperature, the excess acid was removed with methanol using a nitrogen blower. After repeating this process three times, the residue was dissolved in 500 μL of distilled water and centrifuged at 5000× *g* for 10 min. Subsequently, 160 μL of the supernatant was mixed with 160 μL of 0.3 mol/L NaOH and 400 μL of 0.5 mol/L PMP, and the mixture was incubated at 70 °C for 60 min. After cooling to 25 °C, 200 μL of 0.3 mol/L HCl was added to stop the reaction. The mixture was then extracted three times with 0.8 mL of chloroform. The supernatant was collected and filtered through a 0.45 μm membrane. Finally, 10 μL of the aqueous phase was analyzed using an Agilent 1260 series HPLC equipped with an Agilent Eclipse XDB-C18 column (5 μm, 4.6 mm × 250 mm) and a VWD detector set at 254 nm. The mobile phase consisted of 0.1 mol/L ammonium acetate and acetonitrile (83:17, *v*/*v*) at a flow rate of 1.0 mL/min, with the column temperature maintained at 30 °C.

### 3.5. Methylation Analysis

The methylation analysis was performed based on a previously described method with slight modifications [53]. Initially, 5 mg of PJ3-1 was dissolved in 3 mL of anhydrous dimethyl sulfoxide (DMSO), followed by adding 20 mg of sodium hydride (NaH). Methyl iodide (1 mL) was then added dropwise into the mixture in an ice bath with nitrogen gas used to expel the air. The reaction was continued for 1 h and then terminated by adding 1 mL of distilled water. The completion of methylation was confirmed when the hydroxyl absorption peak disappeared from the FT-IR spectrum. Subsequently, 2 mg of the methylated product was redissolved in 2 mL of 2 mol/L TFA and sealed at 105 °C for 6 h. Afterwards, the hydrolysate was reduced with 10 mg of sodium borohydride (NaBH_4_) for 2 h, followed by acetylation with acetic anhydride–pyridine at 100 °C for 2 h. Finally, the partially methylated alditol acetates (PMAAs) were dissolved in 3 mL of chloroform and extracted three times with 3 mL of ultrapure water, which were then redissolved in 100 μL of chloroform after drying by nitrogen flow and analyzed using a TRACE 1300-ISQ instrument (Thermo Scientific, Waltham, MA, USA) equipped with a DB-225 fused silica capillary column (0.25 mm × 30 m, 0.25 μm, Agilent Technologies).

### 3.6. NMR Analysis

PJ3-1 (60 mg) was taken to undergo hydrogen–deuterium exchange for three times with 600 μL of 99.9% D_2_O and then performed at 25 °C on an Agilent DD2 500 MHz NMR spectrometer, including ^1^H NMR, ^13^C NMR, ^1^H–^1^H COSY, TOCSY, HSQC, HMBC and NOESY experiments. MestReNova (V12.0.3, Mestrelab Research, Santiago de Compostela, Spain) software was used for the NMR data analysis. Acetone was used as the internal standard (^1^H 2.225 ppm, ^13^C 31.07 ppm).

### 3.7. Determination of Antioxidant Activities In Vitro

#### 3.7.1. DPPH Free Radical Scavenging Activity

DPPH radical scavenging activity of PJ3-1 was measured according to the published procedure [54]. A total of 100 μL of 0.4 mmol/L of DPPH–ethanol solution and 100 μL of the PJ3-1 solutions (containing 0.125–4.0 mg/mL) were mixed in 96-well plates and then kept in the dark at room temperature. Afterwards, the absorbances were estimated using a microplate reader (Biotek ELx808) at a 517 nm wavelength. The DPPH radical scavenging activity was calculated by the following formula:

$$\mathrm{DPPH}\;\mathrm{scavenging}\;\mathrm{activity}\;(\%)\;=\;(1-\frac{A_{s}-A_{0}}{A_{b}})\times 100$$

where A_s_ is the absorbance for the PJ3-1 solution with DPPH-ethanol solution; A_0_ is the absorbance for the PJ3-1 solution without DPPH; and A_b_ is the absorbance of the mixture of distilled water with the DPPH–ethanol solution.

#### 3.7.2. Hydroxyl Radical Scavenging Activity

Hydroxyl radical scavenging activity was detected as per a previously published method [55]. Specifically, the PJ3-1 solution at various concentrations (0.125-4.0 mg/mL), 0.75 mmol/L 1,10-phenanthroline, 0.75 mmol/L FeSO_4_ and 0.01% H_2_O_2_ were blended at the ratio of 1:1:1:1 and then placed in the dark at 37 °C for 1 h. Ascorbic acid was used as a positive control. The absorbance was measured at 536 nm. The hydroxyl radical scavenging activity was calculated according to the following formula:

$$\mathrm{Hydroxyl}\;\mathrm{radical}\;\mathrm{scavenging}\;\mathrm{activity}\;(\%)\;=\;(1-\frac{A_{s}-A_{0}}{A_{0}-A_{b}})\times 100$$

where A_s_ represents the absorbance of PJ3-1; A_b_ represents the absorbance of the distilled water replaced by PJ3-1; and A_0_ represents the absorbance of the distilled water replaced by PJ3-1 and H_2_O_2_. Vc serves as the positive control.

#### 3.7.3. Scavenging Capacity of Superoxide Anion

PJ3-1 solutions at various concentrations (0.125–4.0 mg/mL) were prepared for the assay. A mixture of 50 μL of the sample solution, 50 μL of 300 μmol/L nitroblue tetrazolium (NBT) solution, 50 μL of 963 μmol/L nicotinamide adenine dinucleotide (NADH) solution and 500 μL of 0.01% (*v*/*v*) hydrogen peroxide (H_2_O_2_) was incubated in the dark for 5 min at room temperature. The absorbance of the mixture was then measured spectrophotometrically at 569 nm. The superoxide anion scavenging capacity was calculated using the following formula:

$$\mathrm{Scavenging}\;\mathrm{capacity}\;\mathrm{of}\;\mathrm{superoxide}\;\mathrm{anion}\;(\%)\;=\;(1-\frac{A_{s}-A_{0}}{A_{b}})\times 100$$

where A_s_ is the absorbance of the PJ3-1 solution; A_0_ is the absorbance of the PJ3-1 solution in distilled water instead of the NBT solution; and A_b_ is the absorbance of the Blank control (PJ3-1 solution replaced by distilled water).

#### 3.7.4. ABTS Scavenging Ability

The ABTS assay was conducted following a previously described method [56]. A total of 14 mmol/L ABTS solution was prepared and allowed to stand at 25 °C for 12 h. The working solution of ABTS was then made by diluting the stock solution with PBS until the absorbance reached 0.7 ± 0.02 at 734 nm. Subsequently, 500 μL of the PJ3-1 solution was mixed with 2 mL of the ABTS working solution and incubated in the dark for 10 min at room temperature. The absorbance of the reaction mixture was measured at 734 nm to determine the scavenging activity, calculated as follows:

$$\mathrm{ABTS}\;\mathrm{scavenging}\;\mathrm{ability}\;(\%)\;=\;(1-\frac{A_{s}-A_{0}}{A_{b}})\times 100$$

where A_s_ is the absorbance of the PJ3-1 solution; A_0_ is the absorbance of the PJ3-1 solution without ABTS solution; and A_b_ is the absorbance of distilled water without PJ3-1 solution.

#### 3.7.5. Ferrous-Ion-Chelating Ability

A total of 100 μL of PJ3-1 at various concentrations was mixed with 50 μL of FeSO_4_ (0.125 mmol/L) and 50 μL of phenoxazine (1 mmol/L). After incubation for 10 min at 37 °C, the absorbance values of the mixtures were measured at 562 nm. Ascorbic acid served as the positive control. The Fe^2+^-chelating activity was calculated using the following formula:

$$\mathrm{Scavenging}\;\mathrm{rate}\;(\%)\;=\;(1-\frac{A_{1}-A_{2}}{A_{0}})\times 100$$

where A_0_ is the absorbance of the mixture without PJ3-1 (replaced by distilled water); A_1_ is the absorbance of the reaction solutions; and A_2_ is the absorbance of the sample alone.

#### 3.7.6. Reducing Power Assay

The reducing power of PJ3-1 was measured according to the procedure described previously [57]. Different concentrations of the PJ3-1 solution (0.125–4.0 mg/mL) were prepared, and 1 mL of each solution was mixed with 2.5 mL of 0.2 mol/L sodium phosphate buffer (pH 7.0) and 1 mL of 1% potassium ferricyanide. The mixture was incubated in a water bath at 50 °C for 20 min, and then 2.5 mL of 10% (*v*/*v*) trichloroacetic acid was added after cooling to room temperature. The mixture was centrifuged at 4000× *g* at room temperature for 10 min. The collected supernatant (2 mL) was diluted with 2 mL of deionized water and then mixed with 400 μL of 0.1% (*w*/*v*) ferric chloride.

Reducing power = A_1_ − A_2_

where A_1_ is the absorbance of the polysaccharides, and A_2_ is the absorbance of the distilled water.

### 3.8. α-Glucosidase-Inhibitory Activity Assay

The α-glucosidase-inhibitory activity was assessed following previously reported methods with slight modifications [48]. A solution of 750 U of α-glucosidase was prepared in 2 mL of 0.01 mol/L PBS (pH 6.8), maintaining the enzyme concentration at 0.5 U/mL. The assay mixture consisted of 90 μL of PJ3-1 at various concentrations (0.08, 0.16, 0.32, 0.64, 1.25, 2.50, and 5.00 mg/mL), 100 μL of α-glucosidase (0.5 U/mL), and 20 μL of p-nitrophenol-D-glucopyranoside (pNPG, 7.5 mmol/L) substrate, which was incubated at 37 °C for 30 min. Acarbose was used as the positive control. The absorbance was measured at 405 nm using a microplate reader (Biotek ELx808, BioTek Instruments, Inc. Winooski, VT, USA). The inhibition rate was calculated using the following formula:

$$\mathrm{Inhibition}\;\mathrm{rate}\;(\%)\;=\;(1-\frac{A_{1}-A_{2}}{A_{0}})\times 100$$

where A_0_ is the absorbance of PJ3-1 or acarbose with PBS; A_1_ is the absorbance of the mixture solution with the sample and pNPG; and A_2_ is the absorbance of the sample without pNPG.

### 3.9. HepG2 Cell Culture

HepG2 cells were purchased from the Chinese Academy of Science (Beijing, China). The cells were cultured in a Petri dish as a monolayer at 37 °C in a humidified atmosphere containing 5% CO_2_. The medium was DMEM containing 10% FBS and 1% penicillin–streptomycin, and the culture medium was changed every 2 days. The cells were subjected to subculture based on their growth status. The cells were rinsed twice with phosphate-buffered saline (PBS), digested with 0.25% trypsin for 3 min, followed by the addition of complete medium to terminate enzymatic digestion and gentle resuspension of the cells. The cell suspension was transferred to 10 mL centrifuge tubes and centrifuged at 3000 rpm for 5 min; the supernatant was carefully discarded, and the resulting cell pellet was gently pipetted with fresh complete medium. Finally, the cell suspension was adjusted to a final density of 5 × 10^5^ cells per milliliter and reserved for subsequent use.

### 3.10. Induction of the Insulin Resistance (IR) Phenotype in HepG2 Cells

To develop and characterize an IR HepG2 cellular model, the effect of different concentrations of glucosamine was assessed in HepG2 cells in terms of intracellular glucose uptake [58,59]. For this procedure, cells were inoculated into 96-well cell culture plates at a volume of 100 μL per well, and maintained overnight at 37 °C in a humidified 5% CO_2_ incubator. Following overnight culture, the conditioned medium was aspirated, each well was rinsed thoroughly three times with phosphate-buffered saline (PBS), and 100 μL of glucose-free DMEM medium was added to each well. Then, treated with glucosamine at concentrations of 6.00, 12.00, 18.00 and 24.00 mmol/L. Cells were cultured in normal medium for 6, 12, 18, 24 and 48 h, respectively. Subsequently, the culture supernatant was aspirated and discarded, 100 μL of glucose-free DMEM medium containing 100 nmol/L insulin was added to each well, and the cells were incubated for a further 2 h. After incubation, the glucose concentration in the culture medium was determined. The experiment was performed in triplicate.

### 3.11. Detection of IR-HepG2 Cell Viability by MTT

The cell viability assay for the PJ3-1 was evaluated via the MTT method, as described by Gong et al. [60]. Following successful establishment of the insulin-resistant HepG2 cell model, PJ3-1 was added to IR-HepG2 cells at varying concentration (50, 100, 200, 400 μg/mL). After incubation for 24 h, the culture medium was discarded and 100 μL of MTT (1 mg/mL) was added. Following a 4 h incubation, the MTT solution was removed, and DMSO was added to dissolve the formazan crystals. The absorbance at 570 nm was then measured using a microplate reader (SpectraMax ID3, San Jose, CA, USA).

### 3.12. Glucose Uptake Assay

After successful establishment of the IR-HepG2 cell model, the test samples were diluted to the required concentrations with culture medium, added to the IR-HepG2 cells, and incubated for 12 h. HepG2 cells under normal culture conditions served as the Blank control group, while IR-HepG2 cells treated with PBS buffer in replacement of the test samples were used as the Model control group. IR-HepG2 cells treated with 200 μg/mL metformin were used as the positive control, and IR-HepG2 cells treated with PJ3-1 at 50, 100 and 200 μg/mL were used as the administration group. The glucose level in the culture supernatant was subsequently determined using a glucose assay kit (Beyotime Institute of Biotechnology, Shanghai, China).

### 3.13. Measurement of Anti-Oxidase and Oxidation Products

The cells were grouped and treated as described in Section 3.11. After washing three times with PBS, cells were harvested by trypsinization. The activities of intracellular antioxidant enzymes (superoxide dismutase, SOD; catalase, CAT) and the level of oxidative product malondialdehyde (MDA) were determined using corresponding commercial kits according to the manufacturer’s instructions (Beyotime Institute of Biotechnology, Shanghai, China). In addition, the intracellular total protein content was quantified with a BCA protein assay kit (Beyotime Institute of Biotechnology, Shanghai, China). MDA content and the relative activities of SOD and CAT were normalized to the total protein content.

### 3.14. Statistical Analysis

Data analyses were performed using Origin 8.0. The results are expressed as the mean ± standard deviation. Data were analyzed by one-way ANOVA and Tukey’s test. *p* < 0.05 was considered statistically significant.

## 4. Conclusions

In summary, the present study successfully obtained an exopolysaccharide PJ3-1 from the mangrove endophytic fungus *Penicillium janthinellum* N29, which containing GlcN, GlcNAc, Glc and GlcA. PJ3-1 is a straight-chain heteropolysaccharide. Interestingly, GlcNAc was first discovered in *Penicillium janthinellum* N29. PJ3-1 can significantly inhibit α-glucosidase activity and alleviate insulin resistance in vitro. It exerts potent antioxidant effects in a dose-dependent manner by mitigating lipid peroxidation and upregulating the activities of endogenous antioxidant enzymes, and it also exhibits free radical scavenging activity in vitro. The hypoglycemic and antioxidant activities of PJ3-1 are probably attributed to the presence of N-acetylglucosamine linked via 1,4-glycosidic bonds and uronic acid in its structure. Therefore, PJ3-1 has great potential to be developed into high-quality antioxidants and hypoglycemic agents and presents promising application prospects in the food and pharmaceutical industries.

## Figures and Tables

**Table 1:** Results of the methylation analysis of PJ3-1.

Methylated Alditol Acetate	Molar Percent Ratio (%)	Linkage Pattern
1,2,5-tri-*O*-acetyl-1-3,4,6-tri-*O*-methyl-glucitol	50.7	→2)-Glc*p*-(1→
1,4,5-tri-*O*-acetyl-2-(acetylmethylamino)-2-deoxy-3,6-di-*O*-methyl-glucitol	31.3	→4)-Glc*p*N-(1→
1,5,6-tri-*O*-acetyl-2-(acetylmethylamino)-2-deoxy-3,4-di-*O*-methyl-glucitol	17.9	→6)-Glc*p*N-(1→

**Table 2:** ^1^H and ^13^C chemical shifts of PJ3-1.

Sugar Residue	Chemical Shifts (ppm) ^a^					
	H1/C1	H2/C2	H3/C3	H4/C4	H5/C5	H6/C6
→2)-α-D-Glc*p*-(1→	5.60/96.4	4.33/81.2	4.18/69.2	4.02/70.0	nd	3.93/60.5
→4)-α-D-Glc*p*A-(1→	5.42/100.6	4.31/74.9	4.03/75.4	3.95/76.3	3.74/71.6	nd
→6)-α-D-Glc*p*NAc-(1→	5.37/97.4	4.24/50.8	3.94/72.6	3.73/71.0	3.87/75.1	4.21/64.6
β-D-Glc*p*-(1→	4.76/96.3	4.09/68.3	3.92/69.3	3.81/71.4	3.66/68.9	4.07/60.4
→4)-β-D-Glc*p*N-(1→	4.66/102.0	4.35/49.4	3.98/72.9	3.88/80.1	3.70/71.2	nd

^a^ Spectra were performed on an Agilent DD2 500 MHz NMR spectrometer. Chemical shifts are referenced to internal acetone at 2.225 ppm for ^1^H and 31.07 ppm for ^13^C. Glc*p*: glucopyranose. nd means not detected.

## Data Availability

Data are contained within the article or Supplementary Materials.

## Supplementary Materials

The following supporting information can be downloaded at: https://www.mdpi.com/article/10.3390/md24080290/s1, Figure S1. FT-IR spectra of the permethylated polysaccharides of PJ3-1; Figure S2. Total ion current chromatograms of PJ3-1; Figure S3 mass spectra of the peaks a–c; Figure S4 Full range NMR spectra of PJ3-1.
