# Supplementary material for: Structural Characterization and Biological Evaluation of a Novel GlcNAc-Bearing Exopolysaccharide from the Mangrove Endophytic Fungus Penicillium janthinellum N29
Source: Mar Drugs. 2026 Aug 21;24(8):290. doi: 10.3390/md24080290 (PMC13514933; doi:10.3390/md24080290)
Supplement: Supplementary file 1 [file marinedrugs-24-00290-s001.zip › marinedrugs-4479242-supplementary.pdf]

Supplementary material

Structural Characterization and Biological Evaluation of a Novel GlcNAc-bearing Exopolysaccharide from the Mangrove Endophytic Fungus *Penicillium janthinellum* N29

Zhuling Shao [1]*, Luya Wang [1] and Xin Wang [1]

[1] School of Food and Biological Engineering, Yantai Institute of Technology, Yantai, 264003, China
* Correspondence: shaozhuling@stu.ouc.edu.cn

Contents

Figure S1. FT-IR spectra of the permethylated polysaccharides of PJ3-1
Figure S2. Total ion current chromatograms of PJ3-1
Figure S3 mass spectra of the peaks a–c
Figure S4 Full range NMR spectra of PJ3-1.

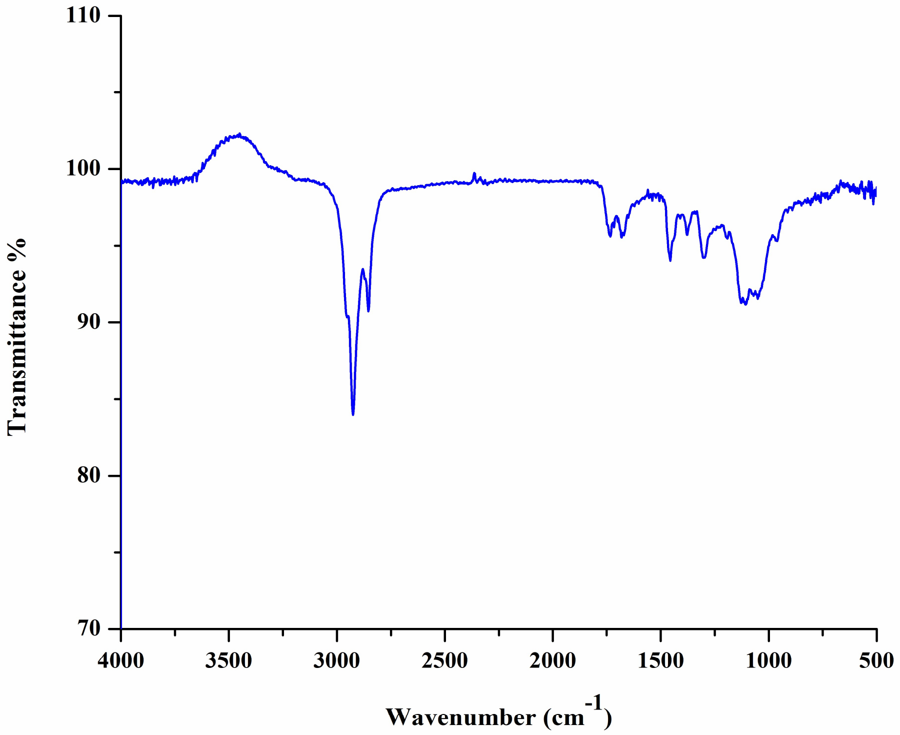

Figure S1. FT-IR spectra of the permethylated polysaccharides of PJ3-1

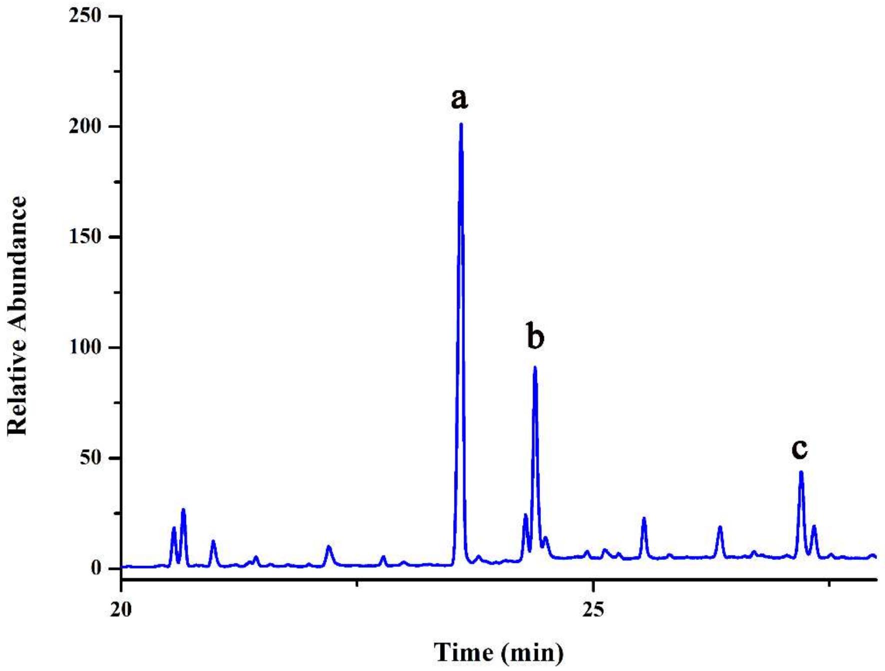

Figure S2. Total ion current chromatograms of PJ3-1

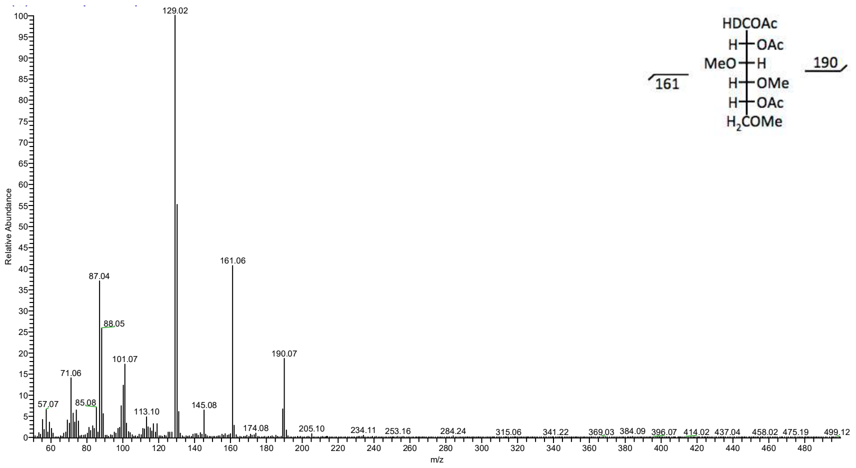

(a) 1,2,5-Tri-O-acetyl-1-3,4,6-tri-O-methyl-glucitol

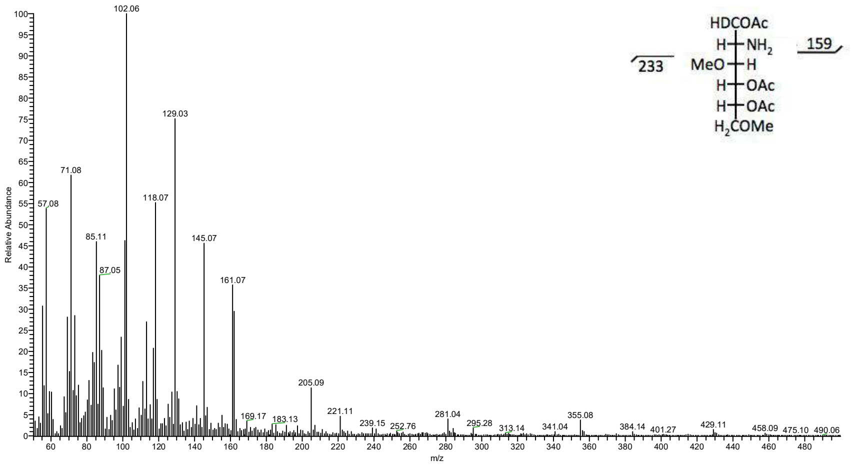

(b) 1,4,5-Tri-O-acetyl-2-(acetylmethylamino)-2-deoxy-3,6-di-O-methyl-D-glucitol

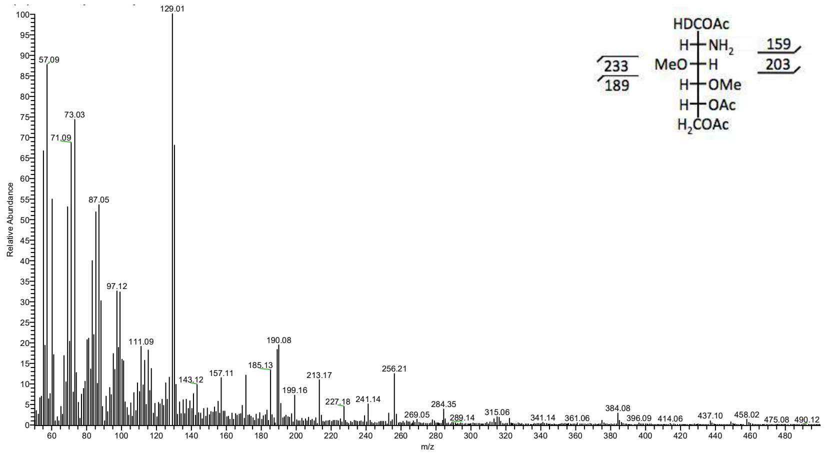

(c) 1,5,6-Tri-O-acetyl-2-(acetylmethylamino)-2-deoxy-3,4-di-O-methyl-D-glucitol

Figure S3 mass spectra of the peaks a–c

^1H-^1H COSY

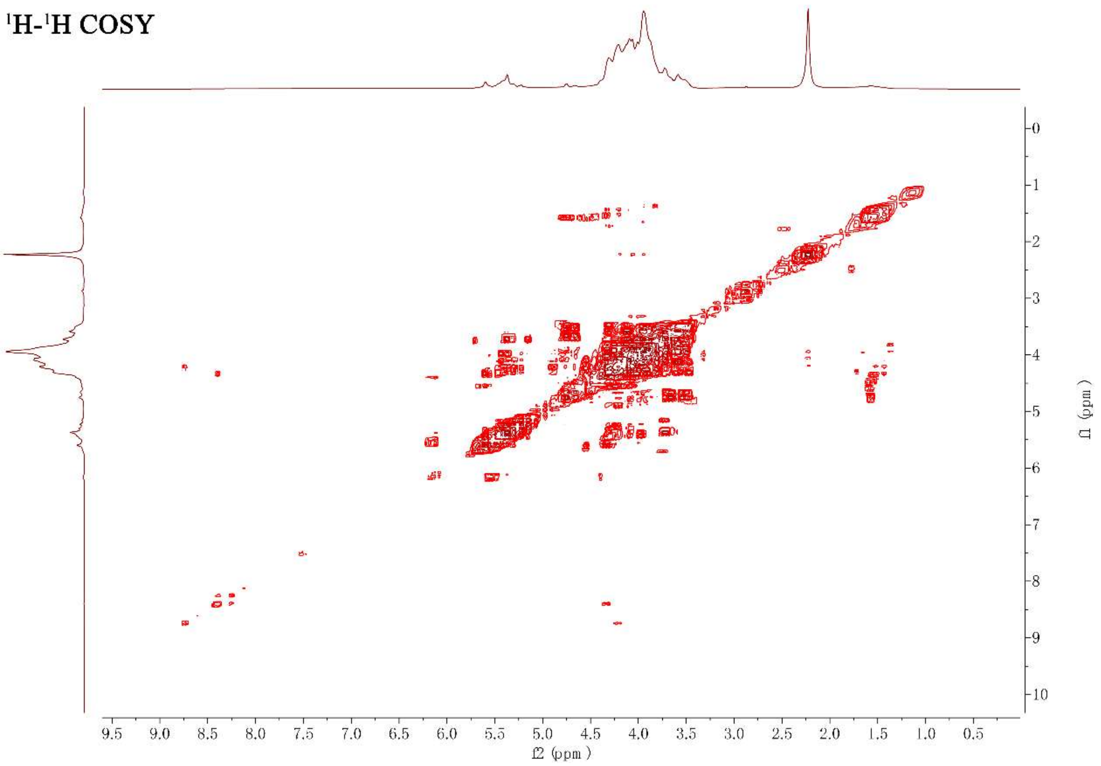

^1H-^{13}C HSQC

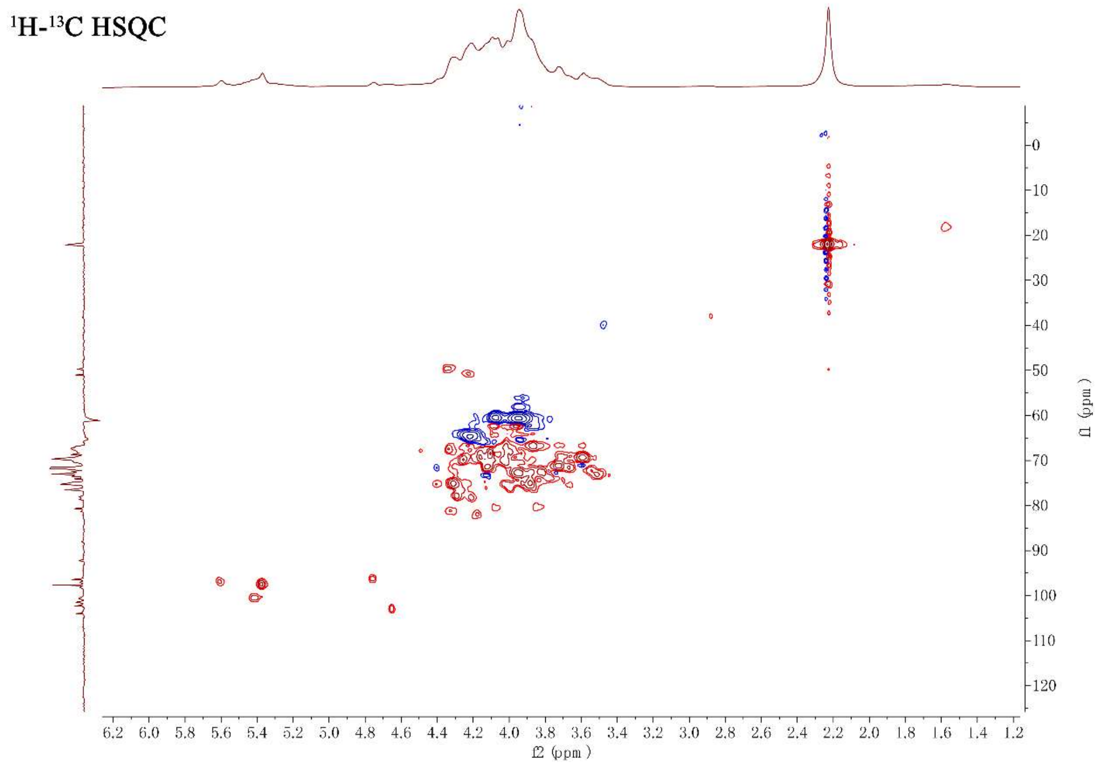

^{1}H-^{1}H NOESY

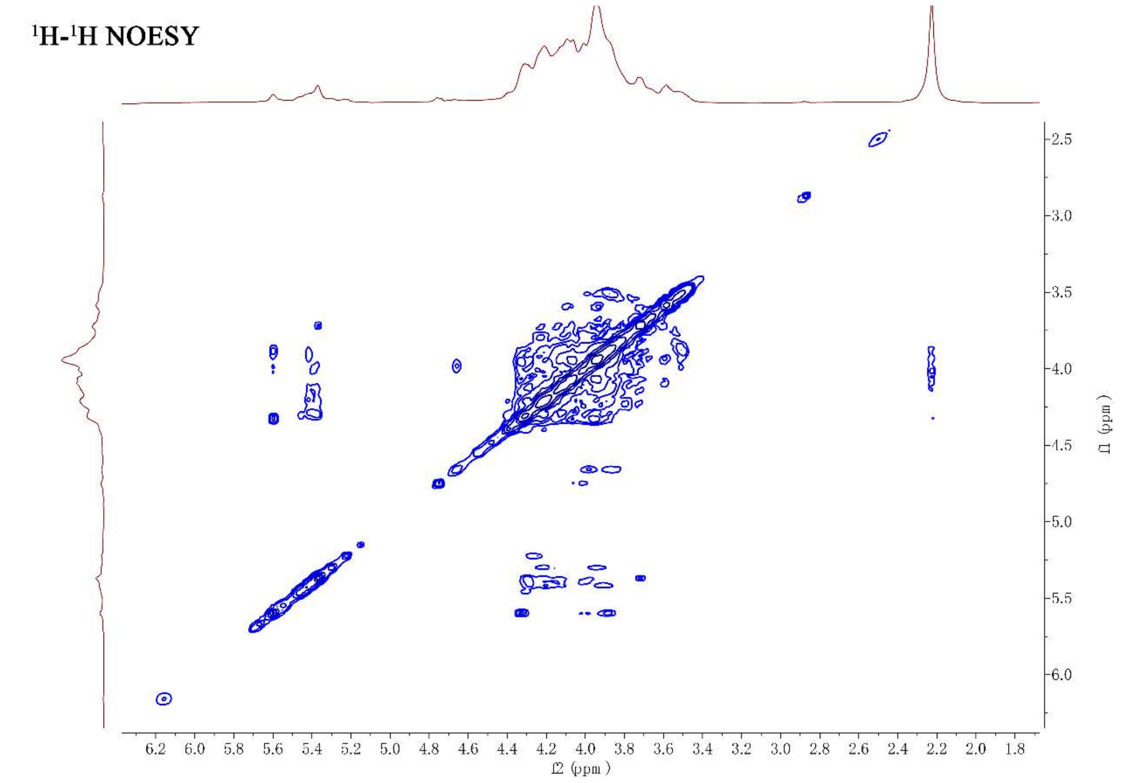

^{1}H-^{1}H TOCSY

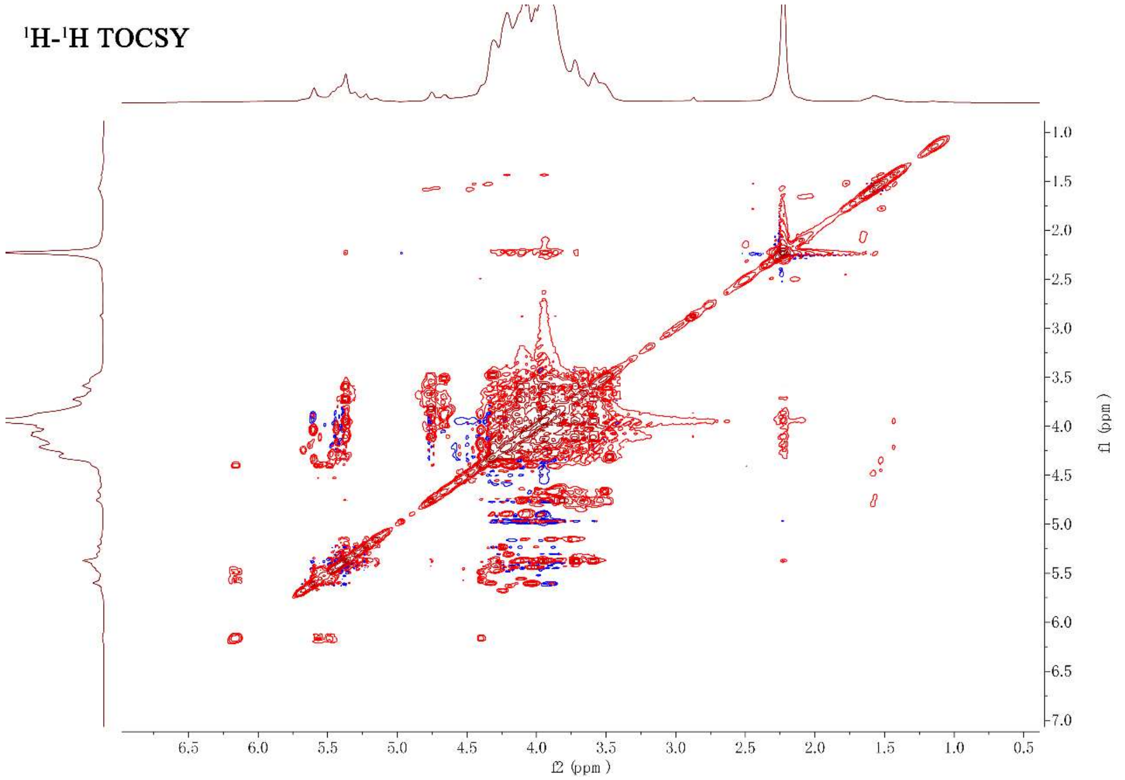

Figure S4 Full range NMR spectra of PJ3-1.
